# Editorial to Special Issue

**DOI:** 10.3390/children9081178

**Published:** 2022-08-05

**Authors:** Sebastian Kwiatkowski

**Affiliations:** Department Obstetrics and Gynecology, Pomeranian Medical University in Szczecin, 70111 Szczecin, Poland; sebastian.kwiatkowski@pum.edu.pl

The definition of preeclampsia has been subject to dramatic changes over the past twenty years. It is now recognized as a syndrome resulting from placental dysfunction and leading to a number of pathological processes such as excessive inflammation, oxidative stress, and impaired angiogenesis, thus causing multiorgan endothelial damage and coagulation defects. Its clinical presentation can be quite diverse and involves both the maternal and fetal systems. Currently, the clinical features of preeclampsia are implicated as including the typical picture of hypertension and proteinuria, as well as other organ dysfunctions such as impaired liver and kidney function, pulmonary edema, and blood–brain barrier dysfunction with symptomatic eclampsia. As for the child, there are symptoms of fetal–placental dysfunction manifested by fetal growth restriction, placental abruption, and abnormal umbilical artery flow indices [1,2,3]. All of these changes raise the legitimate question of whether the current name, although widely recognizable, perhaps requires some adjustment [4].

The new definition has fundamentally changed the incidence of cases classified as preeclampsia from 3% to as much as 10% (according to some current reports) of all pregnancies, although obviously, the degree of severity varies, with the most severe cases still not exceeding 1.5% [5].

Preeclampsia is thus, along with premature rupture of membranes and so-called spontaneous rupture of membranes, the main cause of preterm birth. It is certainly predominant in cases of iatrogenic onset. A large proportion of infants born to preeclamptic mothers are born prematurely and unfortunately carry the associated risks.

Where placental insufficiency is the primary cause of preeclampsia, apart from the features of immaturity, there are also other adverse aspects to fetal development. The adverse environment in which the fetus grows, with a permanently disturbed fetal–placental circulation, leads to impaired development occurring far earlier than in the case of spontaneous preterm birth. Almost 80% of early onset preeclampsia cases are accompanied by fetal growth restriction [6]. A statistically lower birth weight is the first risk factor (and one of the main ones) implying a poorer prognosis and a higher incidence of complications.

Many publications to date have shown that fetuses with growth restriction are at risk of significantly worse postnatal outcomes compared withpreterm infants born at a similar gestational age. These adverse outcomes include a higher incidence of intraventricular hemorrhage and necrotizing enterocolitis, longer stays in intensive care units, and many others [7]. Hypotrophic children are also known to more frequently develop chronic diseases, both of a metabolic nature, such as diabetes and dyslipidemias, and cardiovascular conditions. They are at risk ofmuch higher incidence and earlier onsets of hypertension, myocardial infarctions, and coronary artery disease [8].

Fetuses that develop under ischemic conditions in the placental compartment tend to adapt their metabolism and demonstrate specific energy-saving mechanisms. Congenital insulin resistance is considered one of the manifestations of the intrauterine-sparing effect, which brings about consequences in adult life [9].

Preeclampsia is a very diverse clinical syndrome that develops at different weeks of gestation, a fact that has a major impact on the condition of the neonate. An interesting question, which unfortunately has not been studied in sufficient detail yet, is how the severity of the maternal condition, especially in cases of late-onset preeclampsia or preeclampsia developing at the physiological term of birth, affects the postnatal outcomes in the neonate, and whether late-onset preeclampsia has an equally significant impact on the long-term health complications in these babies. Another separate issue is their actual birth weight and, thus, the degree of growth restriction. Recent reports show that intrauterine weight loss is a very adverse prognostic factor [10]. Preeclampsia also carries an increased risk of emergency obstetric interventions, including emergency Cesarean section, known to statistically increase the risk of developing complications. Currently, the clinical picture also includes placental abruption, capable of dramatically worsening the neonate’s birth status regardless of their gestational age.

There are many challenges ahead and many questions that we haveyet to find the right answers to. In the meantime, here is the latest excellent issue of our journal devoted to the impact of preeclampsia on preterm neonates. I very much hope that some of the doubts that we all share will, thanks to the papers published here, be dispelled and that your curiosity will be satisfied.
